# Skeletal muscle index is an independent predictor of early recurrence in non-obese colon cancer patients

**DOI:** 10.1007/s00423-020-01901-3

**Published:** 2020-06-05

**Authors:** Dagmar Schaffler-Schaden, Christof Mittermair, Theresa Birsak, Michael Weiss, Tobias Hell, Gottfried Schaffler, Helmut Weiss

**Affiliations:** 1 Institute of General Practice, Family Medicine and Preventive Medicine, Paracelsus Medical University, Strubergasse 21, 5020 Salzburg, Austria; 2 Department of Surgery, St. John of God Hospital, Kajetanerplatz 1, 5020 Salzburg, Austria; 3 Department of Surgery, St. John of God Teaching Hospital of Paracelsus Medical University, Kajetanerplatz 1, 5010 Salzburg, Austria; 4 Department of Mathematics, University of Innsbruck, Technikerstrasse 13, 6020 Innsbruck, Austria; 5 Department of Radiology and Nuclear Medicine, St. John of God Teaching Hospital of Paracelsus Medical University, Kajetanerplatz 1, 5010 Salzburg, Austria

**Keywords:** Sarcopenia, Skeletal muscle index, Prehabilitation, Colon cancer, Body composition

## Abstract

**Purpose:**

Progressive loss (sarcopenia) and fatty infiltration of muscle mass (myosteatosis) are well-established risk factors for an adverse clinical outcome in obese patients. Data concerning non-obese sarcopenic patients in oncologic surgery are scarce and heterogeneous. The aim of this study was to determine the impact of sarcopenia and myosteatosis in non-obese patients with cancer of the right colon on clinical outcome.

**Methods:**

This study comprised 85 patients with a BMI < 30 kg/m^2^, who underwent surgery for right colon cancer in a single center. Skeletal muscle area (SMA), visceral fat area (VFA), and myosteatosis were retrospectively assessed using preoperative abdominal CT images. Univariate und multivariate analysis was performed to evaluate the association between body composition, complications, and oncologic follow-up.

**Results:**

Traditional risk factors such as visceral fat (*p* = 0.8653), BMI (*p* = 0.8033), myosteatosis (*p* = 0.7705), and sarcopenia (*p* = 0.3359) failed to show any impact on postoperative complications or early recurrence. In our cohort, the skeletal muscle index (SMI) was the only significant predictor for early cancer recurrence (*p* = 0.0467).

**Conclusion:**

SMI is a significant prognostic factor for early cancer recurrence in non-obese colon cancer patients. Our study shows that conventional thresholds for sarcopenia and BMI do not seem to be reliable across various cohorts. Target prehabilitation programs could be useful to improve outcome after colorectal surgery.

**Trial Registration:**

DRKS00014655, www.apps.who.int/trialsearch

## Introduction

Sarcopenia and obesity represent major public health problems in the aging population. Therapeutic and preventive strategies include physical exercise or nutritional concepts; however, to date, no evidence-based management recommendations exist. Sarcopenia is commonly defined as a progressive loss of skeletal muscle mass and functional strength. This muscle depletion is often accompanied by intra- and intermyocellular fatty infiltration (myosteatosis). Different methods have been introduced to determine these body composition parameters, such as physical performance tests or image analysis assessment [1]. Current literature appears contradictory due to various cut-off values, heterogeneous study populations, and definitions of body composition parameters in oncologic subjects. Sarcopenia and myosteatosis were identified as prognostic factors in various malignancies and are associated with longer hospital stays, increased toxicity of chemotherapy, as well as postoperative morbidity and mortality [2–4]. These adverse effects have also been demonstrated in patients with colorectal cancer (CRC) [2, 5–10]. On the other hand, a number of studies refer to obesity as a factor of higher morbidity and mortality in CRC patients [11–15]. The combination of sarcopenia, myosteatosis, and obesity seems to be a particularly unfavorable constellation. Since the prevalence of obesity and sarcopenia in industrial countries are increasing, many authors reported the adverse impact of sarcopenic obesity on clinical outcomes in several types of tumors [4, 16, 17]. However, the evidence is scarce so far to sufficiently demonstrate the effect of sarcopenia and myosteatosis in the non-obese colon cancer population.

The objective was to investigate the impact of body composition parameters on clinical outcome in a non-obese, homogeneous population with potentially curable colon cancer of the right colon.

## Materials and methods

### Study population and setting

Eighty-nine non-obese patients (BMI < 30 kg/m^2^) with confirmed cancer of the right colon, who underwent elective surgery with curative intent between 2010 and 2016, were enrolled in the study. Eighty-five patients were included in the final analysis; the exclusion of 4 patients was due to incomplete follow-up data. The protocol differentiated patients with conventional open right colectomy (O-RC, *n* = 31) or minimally invasive right colectomy utilizing a transumbilical single-port approach (SIL-RC, *n* = 54).

Patients were included if the following inclusion criteria were met: age > 18 years, confirmed cancer of the right colon, and available preoperative abdominal CT scan (within 30 days before operation). Patients with solitary hepatic metastases had simultaneous removal and were found eligible for inclusion. Exclusion criteria were benign tumor, emergency surgery, recurrent disease, any BMI ≥ 30 kg/m^2^ or missing follow-up data/CT images.

Data of this cohort study was retrieved retrospectively from a prospectively collected electronic database and included demographic data, American Society of Anesthesiologists (ASA) grade, tumor site and TNM stage according to Union for International Cancer Control (UICC), tumor grade and vessel invasion, blood transfusion, diabetes mellitus, conversion rate, and length of hospital stay. Follow-up was performed according to current oncological guidelines for colorectal cancer. The primary endpoint was defined as any evidence of recurrent disease (locally or distant) within 1 year from the date of operation, and the secondary endpoint was intra- and postoperative complications. Complications were assessed according to the Clavien-Dindo system [18]; grade 3 or higher was considered major complications. Demographic parameters of patients and body composition characteristics are shown in Table 1.

**Table 1:** Characteristics^a^ of patients by gender and type of surgery

	Female						Male			
	Total (*n* = 37)	Open (*n* = 14)	SI (*n* = 23)	Estimate with 95% CI^b^	*p* value^c^	Total (*n* = 48)	Open (*n* = 17)	SI (*n* = 31)	Estimate with 95% CI	*p* value
Age (years)	75 (71–82)	74.5 (68.75–79.25)	75 (71–84)	-1 (−9 to 5)	0.6606	76 (67.5–82)	80 (73–83)	74 (65.5–78.5)	5 (−2 to 10)	0.1152
ASA > 2	11/37 (29.7%)	5/14 (35.7%)	6/23 (26.1%)	0.64 (0.12 to 3.47)	0.713	20/48 (41.7%)	8/17 (47.1%)	12/31 (38.7%)	0.72 (0.18 to 2.79)	0.7603
Diabetes mellitus	5/37 (13.5%)	0/14 (0%)	5/23 (21.7%)	Inf (0.59 to Inf)	0.1346	8/48 (16.7%)	1/17 (5.9%)	7/31 (22.6%)	4.55 (0.5 to 222.98)	0.23
Body composition										
Height (m)	161 (158–166)	162 (158.5–165)	161 (157.5–168)	0 (−5 to 4)	0.9624	175 (172.75–180)	175 (173–178)	176 (172–180.5)	0 (−4 to 3)	0.9741
Weight (kg)	64 (56–69)	64.5 (57.75–67.5)	64 (54–70.5)	0 (−7 to 8)	0.925	80 (71–86)	73 (69–82)	83 (72.5–88.5)	−7 (−14 to −1)	0.0171
BMI (kg/m2)	23.95 (21.88–26.37)	24.02 (22.09–25.92)	23.88 (21.27–26.13)	0.2 (−2 to 2.74)	0.8756	25.49 (23.52–27.51)	23.74 (23.05–25.54)	26.73 (24.54–28.24)	−2.24 (−3.77 to −0.83)	0.0053
Subcutaneous fat (cm2)	156.92 (111.49–200.45)	152.04 (117.6–191.11)	157.87 (111.04–211.77)	−1.01 (−50.86 to 44.69)	0.8894	129.34 (91.94–168.45)	113.04 (80.61–129.94)	138.19 (98.41–170.24)	−21.81 (−53.63 to 6.3)	0.1268
Visceral fat (cm2)	59.44 (35.68–114.01)	87.91 (48.7–118.89)	54.2 (32.27–111.33)	7.89 (−29.75 to 45.26)	0.6538	178.11 (130.36–251.19)	150.57 (114.58–249.19)	181.65 (152.58–262.9)	−32.57 (−92.25 to 16.83)	0.1694
SMI (cm2/m2)	36.89 (33.47–41)	34.75 (32.07–39.4)	38.82 (34.4–41.91)	−3.2 (−6.95 to 0.99)	0.1221	46.94 (43.65–51.08)	42.34 (40.88–46.73)	49.67 (46.3–51.78)	−5.5 (−8.89 to −2.66)	0.0008
Sarcopenia (Martin et. al)	10/37 (27%)	2/14 (14.3%)	8/23 (34.8%)	3.11 (0.49 to 35.42)	0.2603	16/48 (33.3%)	4/17 (23.5%)	12/31 (38.7%)	2.02 (0.47 to 10.56)	0.3503
Sarcopenia (SMI < 1/3-quantile)	14/37 (37.8%)	8/14 (57.1%)	6/23 (26.1%)	0.28 (0.05 to 1.33)	0.0851	18/48 (37.5%)	10/17 (58.8%)	8/31 (25.8%)	0.25 (0.06 to 1.01)	0.0324
Myosteatosis	33.1 (27.4–39.6)	32.35 (23–34.48)	33.5 (27.7–41.8)	−4.24 (−10.5 to 3)	0.2868	32.1 (25.05–38.95)	33.6 (31.1–36.9)	28.1 (23.8–39.2)	4.5 (−3 to 9.4)	0.2073
N positive	19/37 (51.4%)	10/14 (71.4%)	9/23 (39.1%)	0.27 (0.05 to 1.29)	0.0911	15/48 (31.2%)	8/17 (47.1%)	7/31 (22.6%)	0.34 (0.08 to 1.41)	0.1083
M positive	5/37 (13.5%)	5/14 (35.7%)	0/23 (0%)	0 (0 to 0.54)	0.0046	6/48 (12.5%)	3/17 (17.6%)	3/31 (9.7%)	0.51 (0.06 to 4.29)	0.6513
T = 4	3/37 (8.1%)	2/14 (14.3%)	1/23 (4.3%)	0.28 (0 to 5.96)	0.5441	5/48 (10.4%)	3/17 (17.6%)	2/31 (6.5%)	0.33 (0.02 to 3.23)	0.3306
L positive	13/37 (35.1%)	6/14 (42.9%)	7/23 (30.4%)	0.59 (0.12 to 2.92)	0.4948	13/48 (27.1%)	6/17 (35.3%)	7/31 (22.6%)	0.54 (0.12 to 2.45)	0.4983
V positive	3/37 (8.1%)	1/14 (7.1%)	2/23 (8.7%)	1.23 (0.06 to 78.55)	1	5/48 (10.4%)	3/17 (17.6%)	2/31 (6.5%)	0.33 (0.02 to 3.23)	0.3306
Chemotherapy	17/37 (45.9%)	9/14 (64.3%)	8/23 (34.8%)	0.31 (0.06 to 1.44)	0.1014	15/48 (31.2%)	7/17 (41.2%)	8/31 (25.8%)	0.5 (0.12 to 2.12)	0.3364
Complications Dindo ≥ 3	6/37 (16.2%)	2/14 (14.3%)	4/23 (17.4%)	1.26 (0.15 to 15.9)	1	15/48 (31.2%)	4/17 (23.5%)	11/31 (35.5%)	1.77 (0.4 to 9.28)	0.5207
Recurrent disease	11/37 (29.7%)	8/14 (57.1%)	3/23 (13%)	0.12 (0.02 to 0.7)	0.0084	8/48 (16.7%)	4/17 (23.5%)	4/31 (12.9%)	0.49 (0.08 to 3.07)	0.4285

^a^Binary data are presented as no./total no. (%), continuous data as medians (25th–75th percentile). ^b^Odds ratio for binary variables and estimated median difference for continuous variables. ^c^Differences between groups assessed by Fishers` exact test for binary variables and Wilcoxon rank sum test for continuous variables. SI, single incision surgery; SMI, skeletal muscle index

### Surgical technique

Patient management followed a modified enhanced recovery strategy protocol in all study patients. This protocol included thoracic epidural analgesia, avoidance of excess fluid administration and volume overload, a multimodal antiemetic approach, no drainage or nasogastric tube, early progressive mobilization and oral nutrition, early removal of epidural and urinary catheters, and opioid sparing analgesia.

The choice of surgical approach (open or SIL) depended upon the surgeons` preference; all operations were performed by experienced surgeons. Open conventional surgery was performed in patients in supine position via a transverse right abdominal incision following the principles of complete mesocolic excision (CME) with dissection of the respective lymph node basin up to the trunk of Henle. Ileocolic anastomosis was established in a side-to-side stapler technique. Mesentery defects were re-approximated routinely. SIL procedures were performed using disposable devices (SILS-Port™, Medtronic, Dublin, Ireland; GelPort™, Applied Medical, Rancho Santa Margarita, USA; OctoPort™, DalimSurgNET, Frankenman Group, Seoul, Korea) at the umbilical site only. Additional trocars, other than this device, were not necessary in any patient. In all procedures, extra-long optical devices (10 mm diameter, 30° optics) were used. Additional instruments or suspension devices were used in all patients and delivered through the umbilical site to alleviate the exposure of the surgical field. CME and extended lymphadenectomy were carried out according to the open technique.

### Image analysis

The assessment of body composition was performed by two independent investigators on preoperative routine CT scans, after individual training, who were blinded to the patients’ identity. Scans with a strong deviation of values were checked by a senior radiologist. CT scans within 30 days prior surgery were considered suitable for examination. Contrast-enhanced CT images with 2.5–3-mm slice thickness (100–130 kVp, and 150–300 mAS, Emotion 6 or Somatom Definition AS Siemens Healthcare GmbH, Erlangen) at the level of the third lumbar vertebra (L3) were retrieved from the Picture Archiving and Communication System (PACS©) of each patient. Image J (https://imagej.net/ImageJ) as an open source software was used to analyze each single image. Hounsfield Unit (HU) thresholds were used as previously described for skeletal muscle (− 29 to + 150), for subcutaneous fat (− 190 to − 30), and for visceral fat tissue (− 150 to − 50) [19]. Muscle area included psoas muscle, rectus abdominis, quadratus lumborum, erector spinae, abdominal lateral, and oblique muscle. Mean muscle attenuation (HU) representing myosteatosis was assessed at Level L3. The value for the skeletal muscle area was normalized for height (m^2^) to create the skeletal muscle index (SMI, cm^2^/m^2^).

Sarcopenia was defined according to gender and BMI categories (BMI 20.0–29.9 kg/m^2^) as published previously [20]. Additionally, with respect to regional variations, the lowest sex-specific quartile of SMI within the study population was classified as sarcopenia for comparison.

### Statistical analysis

The threshold for statistical significance was considered at a *p* value < 0.05; all tests were two-sided. Potential risk factors for complications were investigated using univariate analysis, namely, Fisher’s exact test, Chi-square tests, the Wilcoxon rank sum test, the Wilcoxon signed rank test, the Kruskal-Wallis test, and logistic univariate regression adjusted for gender in case of body composition parameters. Cox hazards regression stratified by gender for body composition parameters was performed for tumor recurrence. Amendatory, multivariate Cox hazards regression analysis was also conducted with significant univariate predictors for tumor recurrence and incorporating different treatment groups (open versus single-port laparoscopic). The statistical analyses were conducted using the software R (version 3.5.3; http://www.r-project.org).

## Results

Thirty-one patients underwent open resection, and 54 patients SIL surgery. The surgical procedure was completed in all patients. The conversion rate was 5.5% (3/54). Reasons for conversion were tumor size and adhesions in two cases and adhesions in one case. No intraoperative complications were observed. Fifteen major complications (17.6%, Clavien-Dindo ≥ 3) requiring further intervention occurred; for further details, see Table 2. The overall rate of anastomotic leakage was 6/85 (7%); one occurred in the O-RC group (handsewn), and the remaining five occurred in the SIL-RC group (all stapled: four intracorporeally, one extracorporeally).

**Table 2:** Overview of major and minor complications

Complications	Dindo ≥ 3	Data
Anastomotic leakage		6 (7%)
Hemorrhage		4 (4.7%)
Fascia dehiscence		3 (3.5%)
Ileus		1
Abscess		1
	Dindo < 3	
Cardiac decompensation		1
Hematoma/Abscess		3
Pneumonia		2

The prevalences of sarcopenia in total were 30.6% and 32.9% respectively, depending on the assessment method; mean value for myosteatosis (HU) in total was 32.3 (see Table 1). Thirty-day mortality was zero in the entire cohort. The postoperative oncologic follow-up revealed nine (29%) patients with recurrent disease in the O-RC group, and three patients in the SIL-RC group.

**Fig. 1:**
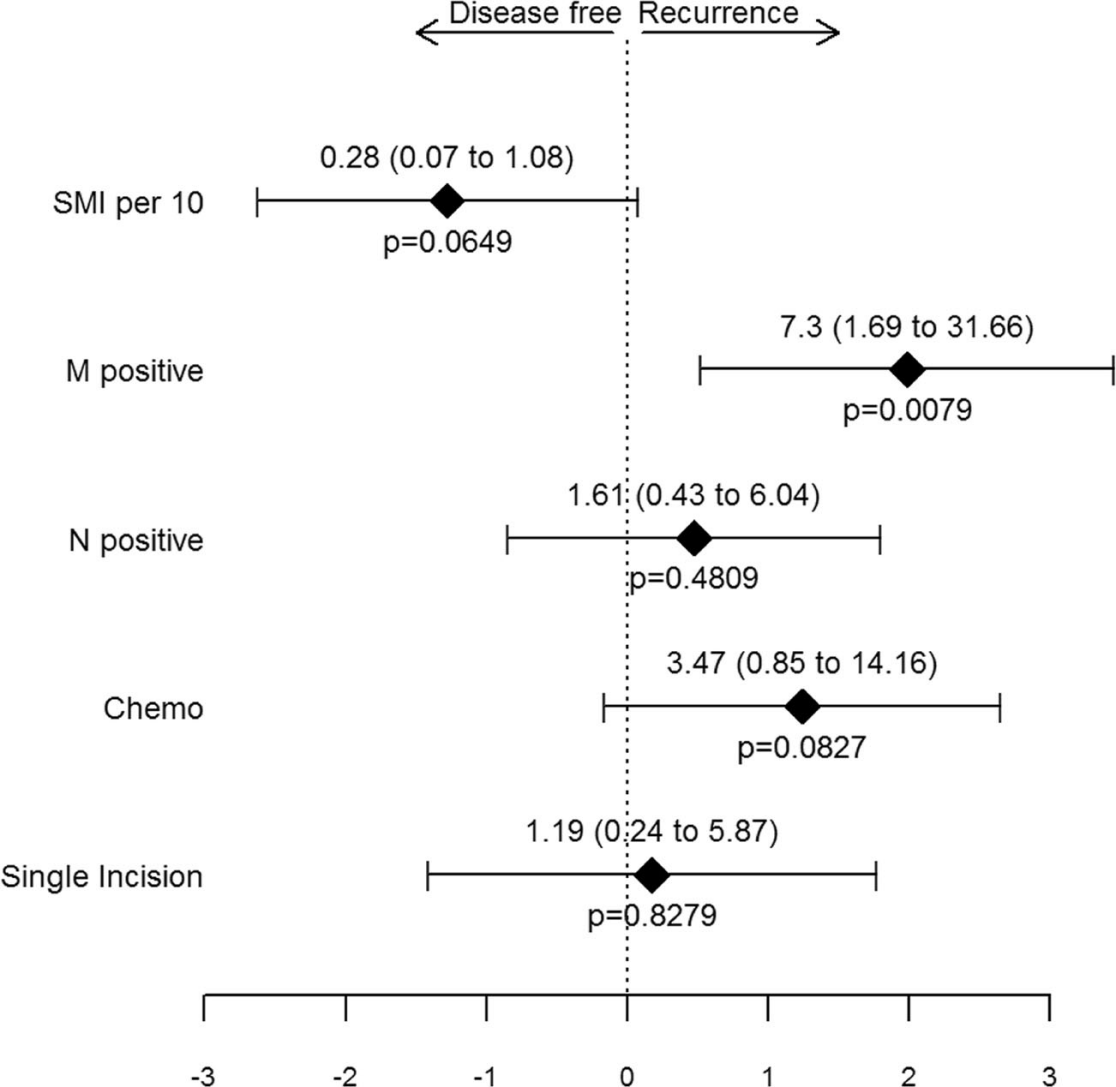
Hazard ratios retrieved from Cox proportional harzards model for survival stratified by gender. Depicted are effect sizes (log hazard ratio); provided are hazard ratios with 95% CIs and corresponding *p* values. Adjusting for M positive, the risk for 1-year-recurrence is comparable (Hazard ratio 0.4 (2.5 to 6.39), *p* = 0.6478) in the open and laparoscopic group

In our study population, none of the traditional body composition parameters (BMI, subcutaneous fat tissue, visceral fat tissue) or myosteatosis had a significant impact on complications or tumor recurrence in univariate or multivariate calculation. With regard to muscle depletion, all parameters were determined by univariate and multivariate analysis. See an overview of results in Table 3. SMI was the only prognostic relevant factor for tumor-free survival in univariate analysis, but was not associated with postoperative complications. In multivariate analysis, the SMI was not significant which can be contributed to the sample size. Prevalent metastatic disease was the only significant negative oncologic predictor. Minimizing the surgical trauma by means of SIL could not show any significant impact during the oncologic follow-up. The risk for 1-year-tumor recurrence was significantly higher for patients who underwent open surgery as compared with single incision (*p* = 0.0241). However, the rate of patients with metastasis (M-positive) was significantly higher in the open surgery group as compared with single incision (8/31 (25.8%) vs. 3/54 (5.5%), *p* = 0.0151). When adjusting the groups for M positive, the risk for 1-year-recurrence is comparable (*p* = 0.6478), (see Fig. 1).

**Table 3:** Characteristics^a^ of patients by complication and recurrence of disease within 1 year

		Complications				One year recurrence of disease	
	Total (*n* = 85)	Complication (*n* = 21)	No complication (*n* = 64)	Estimate with 95% CI^b^	*p* value^c^	Hazard ratio with 95% CI^d^	*p* value
Age (years)	75 (69–82)	74 (61–81)	75 (70.75–82)	−2 (−8 to 3)	0.4659	0.98 (0.93 to 1.03)	0.4611
ASA > 2	31/85 (36.5%)	10/21 (47.6%)	21/64 (32.8%)	0.54 (0.18 to 1.67)	0.2967	1.25 (0.4 to 3.94)	0.7039
Diabetes mellitus	13/85 (15.3%)	2/21 (9.5%)	11/64 (17.2%)	1.96 (0.37 to 19.78)	0.5042	0 (0 to Inf)	0.9976
Body composition							
Height (m)	172 (162–176)	173 (168–178)	171.5 (162–176)	2 (−3 to 7)	0.4323	1.01 (0.92 to 1.1)	0.8679
Weight (kg)	71 (64–83)	72 (65–84)	70 (64–82)	2 (−6 to 8)	0.6067	1.01 (0.95 to 1.06)	0.7801
BMI (kg/m2)	24.8 (22.76–26.78)	24.36 (22.76–26.56)	24.98 (22.95–27.13)	−0.36 (−1.95 to 1.13)	0.6502	1.02 (0.84 to 1.24)	0.8033
Subcutaneous fat (cm2)	138.64 (95.37–180.46)	134.15 (102.42–180.46)	140.51 (95.13–180.63)	−7.51 (−37.97 to 22.71)	0.614	1 (0.99 to 1.01)	0.7492
Visceral fat (cm2)	133.6 (76.36–199.16)	144.32 (93.95–180.83)	127.03 (72.13–203.42)	2.28 (−45.03 to 54.13)	0.9229	1 (0.99 to 1.01)	0.8653
SMI (cm2/m2)	42.45 (37.21–48.07)	46.37 (39.79–47.22)	41.89 (36.17–49.13)	2.7 (−1.79 to 6.01)	0.2559	0.9 (0.81 to 1)	0.0467
Sarcopenia (Martin et. al)	26/85 (30.6%)	7/21 (33.3%)	19/64 (29.7%)	0.85 (0.27 to 2.89)	0.7886	0.2 (0.03 to 1.52)	0.1184
Sarcopenia (SMI < 1/3-quantile)	32/85 (37.6%)	6/21 (28.6%)	26/64 (40.6%)	1.7 (0.53 to 6.08)	0.4379	0.57 (0.19 to 1.78)	0.3359
Myosteatosis	32.3 (25.1–39.1)	30.3 (25.3–40.3)	32.55 (24.8–38.6)	−0.1 (−5.1 to 5.2)	0.9837	1.01 (0.95 to 1.07)	0.7705
N positive	34/85 (40%)	6/21 (28.6%)	28/64 (43.8%)	1.93 (0.61 to 6.89)	0.3056	3.33 (1 to 11.06)	0.0497
M positive	11/85 (12.9%)	2/21 (9.5%)	9/64 (14.1%)	1.55 (0.28 to 15.98)	0.7241	8.66 (2.79 to 26.95)	0.0002
T = 4	8/85 (9.4%)	1/21 (4.8%)	7/64 (10.9%)	2.44 (0.28 to 116.09)	0.6728	2.1 (0.46 to 9.6)	0.3373
L positive	26/85 (30.6%)	6/21 (28.6%)	20/64 (31.2%)	1.13 (0.35 to 4.11)	1	1.63 (0.52 to 5.14)	0.4039
V positive	8/85 (9.4%)	4/21 (19%)	4/64 (6.2%)	0.29 (0.05 to 1.72)	0.0991	2.19 (0.48 to 10.01)	0.3112
Chemotherapy	32/85 (37.6%)	4/21 (19%)	28/64 (43.8%)	3.26 (0.92 to 14.83)	0.0678	5.3 (1.43 to 19.61)	0.0124
Therapy							
Single Incision	54/85 (63.5%)	15/21 (71.4%)	39/64 (60.9%)	0.63 (0.18 to 2)	0.4433	0.25 (0.08 to 0.83)	0.0241

^a^Binary data are presented as no./total no. (%), continuous data as medians (25th–75th percentile). ^b^Odds ratio for binary variables and estimated median difference for continuous variables. ^c^Differences between groups assessed by Fisher s exact test for binary variables and Wilcoxon rank sum test for continuous variables. ^d^Retrieved from Cox proportional hazards regression model (stratified by gender for body composition parameters)

## Discussion

**Fig. 2:**
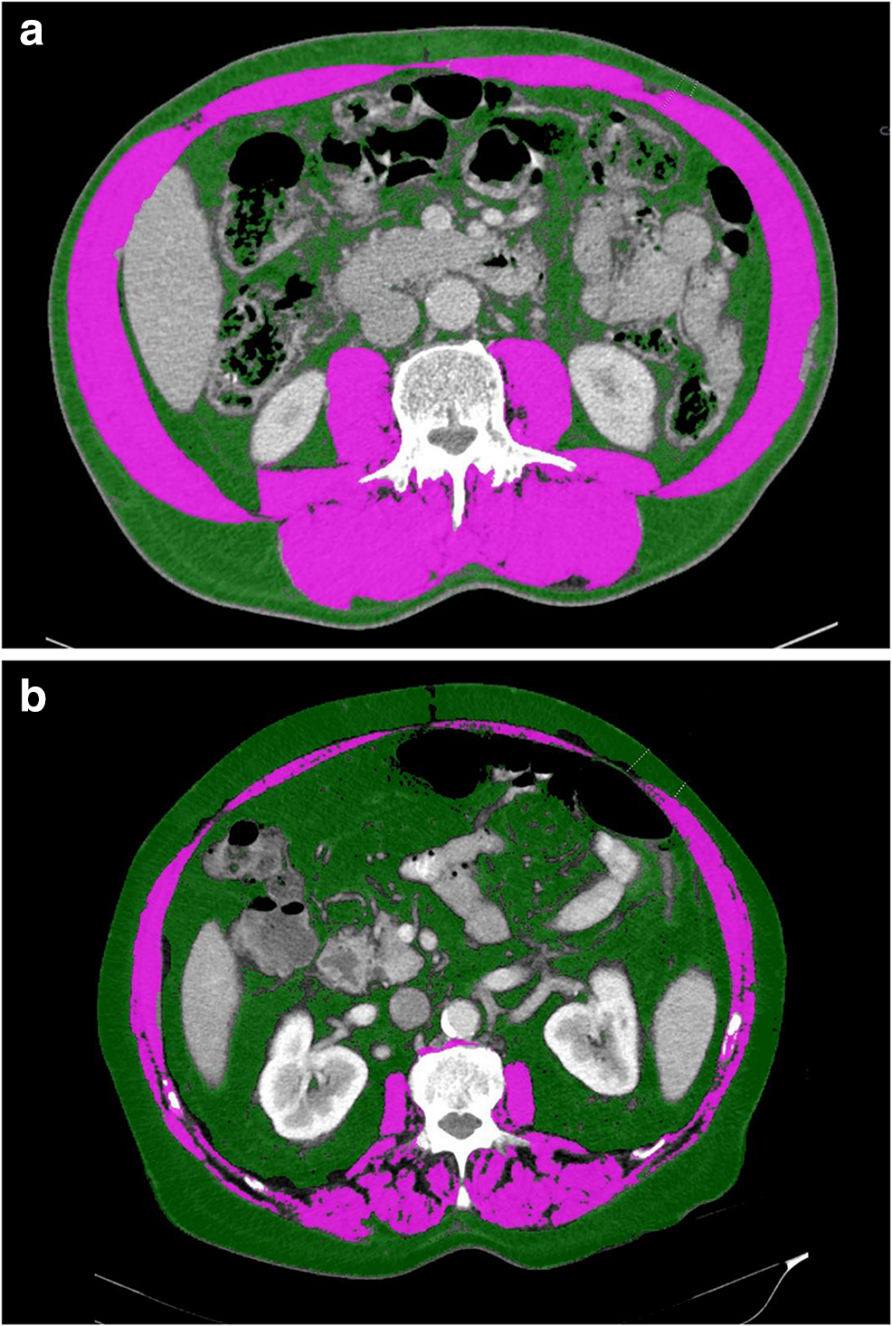
Abdominal CT images of patients with identical BMI (28.7 kg/m^2^), patient A with a SMI of 67.8 cm^2^/m^2^, and patient B with a SMI 35.3 cm^2^/m^2^; green: subcutaneous and visceral fat tissue, pink: skeletal muscle tissue

In this study, the SMI was the only prognostic factor with respect to early recurrence of non-obese colon cancer patients, while all other common risk factors or body composition parameters failed to show any significant impact. Another study including patients with primary operable colorectal cancer yielded similar findings [21]. These results raise the question for the validity of absolute thresholds for sarcopenia. Differences in definitions and the wide range of thresholds for sarcopenia, myosteatosis, and visceral obesity are contributing to remarkable variations and hamper the application of findings in the clinical practice [22]. In a more recent meta-analysis, sarcopenia was found to be a consistent risk factor for major complications after surgery of gastrointestinal tumors independent of the assessment method, but the subgroup analysis for colorectal cancer was not significant. Moreover, evidence of sarcopenia being a risk factor after gastrointestinal tumor resection was rated as very low according to GRADE criteria [23]. Regarding the applied cut-off values, neither sarcopenia nor myosteatosis were associated with cancer recurrence or complications in our cohort, which is in contradiction to many studies suggesting undesirable outcomes in cancer patients related to body composition parameters [9, 17, 24–26]. However, study results are still controversial and only comparable to a limited extent. Geographic and ethnic diversity of study populations related to body composition thresholds have been reported previously [23, 27]. Some authors observed a higher prevalence of postoperative complications in Asian sarcopenic patients with non-metastatic CRC [28]. Regarding several recently published meta-analyses and systematic reviews, it can be determined that a variety of body composition assessment methods, thresholds, and study populations were included. Sun et al. focused on patients with non-metastatic CRC indicating that sarcopenia is a risk factor for postoperative morbidity and mortality. This meta-analysis included six studies from Asia [28]. The reported prevalence of sarcopenia varies between 5 and 79%, depending on the population [2, 29]. Thresholds for one ethnic group are probably not applicable for other groups. Sarcopenia was observed in patients with any BMI and body weight [30], (see Fig. 2). BMI therefore seems not suitable as a predictor for surgical complications as shown in patients with advanced rectal cancer [31]. Obesity combined with muscle depletion (sarcopenic obesity) seems to be a particularly unfavorable condition, as it is linked to a higher morbidity and mortality in patients after cancer surgery [16, 24, 32]. Regarding sarcopenic and visceral obesity*,* various definitions exist as well [17, 33, 34]. Apparently, results are often depending on cut-off levels, assessment methods, and definitions of body composition thresholds. The positive impact of (conventional) laparoscopic surgery on sarcopenic patients has been confirmed, although evidence for the impact of surgical approach (open vs. laparoscopic) related to body composition parameters is scarce [17, 35, 36].The advantages of minimally invasive surgery in colorectal cancer regarding blood loss, length of hospital stay, wound pain, and postoperative complications have been reported in large randomized controlled trials [37]. Due to the small sample size and heterogeneity in the two groups (patients in the open group had more often advanced disease), it was not possible to explore the impact of surgical approach on the clinical outcome in our cohort. Prevention and therapy of sarcopenia and its known adverse effects are currently the major focus of many research projects. Targeted concepts with short-term resistance training and nutritional supplementation have shown promising results in the treatment of sarcopenia [38–40]. Especially physical activity seems to have a positive impact on surgical outcome as a randomized-controlled trial proved—although the preoperative timeframe is usually short [41]. A recent review reported an improved 5-year disease-free survival for colorectal cancer patients undergoing prehabilitation, at least for the subgroup with stage III cancer. No benefit was observed concerning the overall survival [42]. However, there is no evidence regarding which patients will benefit most from an adapted preoperative nutrition assessment and physical exercise training prior to surgery [43]. Nevertheless, preoperative identification of patients with higher operative risk is essential to avoid a complicated course with serious impact on quality of life.

This study has several limitations. Interobserver variability was not assessed for the image evaluation. Furthermore, functional muscle assessment was not included and no long-term outcomes were reported. As we focused on a non-obese population with the same cancer localization, the sample size was limited. The strength of this study is the ethnically homogeneous study population with potentially curable colon cancer located in the right colon, all operated in a single center according to a standardized open or laparoscopic procedure.

In conclusion, a reduced SMI is an important predictive factor for early recurrence of colon cancer. Heterogeneity of assessment methods, study populations, and threshold variations of body composition parameters currently hamper comparability of study results in daily surgical routine. Absolute thresholds for sarcopenia do not seem to be reliable in different settings. The preoperative identification of patients at risk for an unfavorable postoperative course seems to be an important issue. The results of this study should be interpreted with caution, as our cohort did not undergo a specific preoperative nutrition or training program. Well-designed randomized studies could probably help to show the impact of individually tailored prehabilitation programs on the postoperative clinical course in sarcopenic patients.
